# Soil Salinity and pH Drive Soil Bacterial Community Composition and Diversity Along a Lateritic Slope in the Avon River Critical Zone Observatory, Western Australia

**DOI:** 10.3389/fmicb.2019.01486

**Published:** 2019-07-02

**Authors:** Flora J. M. O’Brien, Maya Almaraz, Melissa A. Foster, Alice F. Hill, David P. Huber, Elizabeth K. King, Harry Langford, Mary-Anne Lowe, Bede S. Mickan, Valerie S. Miller, Oliver W. Moore, Falko Mathes, Deirdre Gleeson, Matthias Leopold

**Affiliations:** ^1^Biological Sciences, University of Southampton, Southampton, United Kingdom; ^2^National Center for Ecological Analysis and Synthesis, University of California, Santa Barbara, Santa Barbara, CA, United States; ^3^U.S. Bureau of Reclamation, Denver Federal Center, Denver, CO, United States; ^4^Cooperative Institute for Research in Environmental Sciences, University of Colorado, Boulder, Boulder, CO, United States; ^5^Department of Biological Sciences, Idaho State University, Pocatello, ID, United States; ^6^Department of Marine Chemistry and Geochemistry, Woods Hole Oceanographic Institution, Woods Hole, MA, United States; ^7^Department of Geography, The University of Sheffield, Sheffield, United Kingdom; ^8^UWA School of Agriculture and Environment, The University of Western Australia, Crawley, WA, Australia; ^9^Department of Renewable Resources, University of Alberta, Edmonton, AB, Canada; ^10^School of Earth and Environment, University of Leeds, Leeds, United Kingdom

**Keywords:** soil microbial community, bacteria, laterite, critical zone, Western Australia

## Abstract

Soils are crucial in regulating ecosystem processes, such as nutrient cycling, and supporting plant growth. To a large extent, these functions are carried out by highly diverse and dynamic soil microbiomes that are in turn governed by numerous environmental factors including weathering profile and vegetation. In this study, we investigate geophysical and vegetation effects on the microbial communities of iron-rich lateritic soils in the highly weathered landscapes of Western Australia (WA). The study site was a lateritic hillslope in southwestern Australia, where gradual erosion of the duricrust has resulted in the exposure of the different weathering zones. High-throughput amplicon sequencing of the 16S rRNA gene was used to investigate soil bacterial community diversity, composition and functioning. We predicted that shifts in the microbial community would reflect variations in certain edaphic properties associated with the different layers of the lateritic profile and vegetation cover. Our results supported this hypothesis, with electrical conductivity, pH and clay content having the strongest correlation with beta diversity, and many of the differentially abundant taxa belonging to the phyla Actinobacteria and Proteobacteria. Soil water repellence, which is associated with *Eucalyptus* vegetation, also affected beta diversity. This enhanced understanding of the natural system could help to improve future crop management in WA since the physicochemical properties of the agricultural soils in this region are inherited from laterites via the weathering and pedogenesis processes.

## Introduction

Soils provide a variety of essential ecosystem services which support life above- and below-ground (Smith et al., 2015; Adhikari and Hartemink, 2016). They form part of the Earth’s critical zone (CZ), which spans from the canopy to the bedrock, incorporating a complex network of biogeochemical processes and cycles that sustain terrestrial life (Brantley et al., 2007). Characterizing microbial responses to bottom-up effects of soil physicochemical properties versus top-down vegetation effects is important to understanding CZ architecture and predicting functional changes that may result from disturbance activities (Chorover et al., 2007).

**FIGURE 1:**
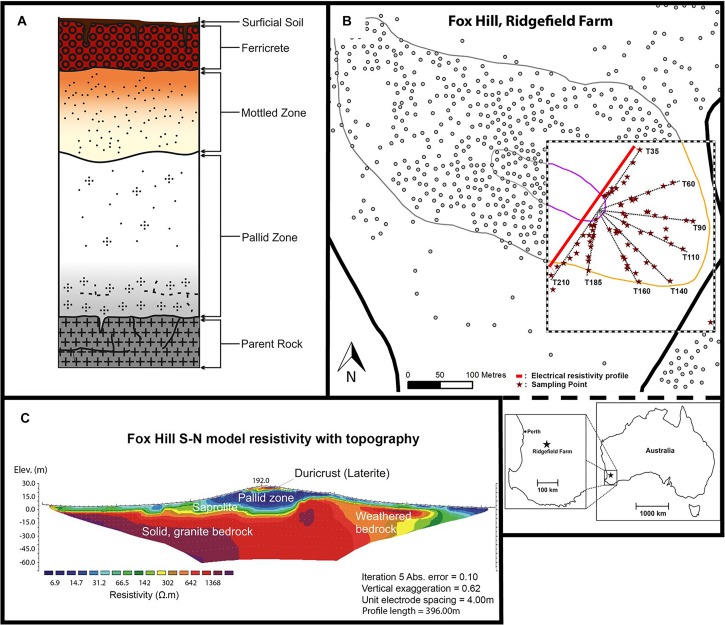
Composition of the lateritic profile **(A)**; site location at Fox Hill **(B)**; and ERT profile across the hill along T35 and T210 **(C)**.

In Western Australia (WA), extensive and deep weathering of Archaean granitic bedrock has resulted in the formation of lateritic soils that cover much of the region, including a 44,000 km^2^ area in the southwest (Gilkes et al., 1973; Anand and Paine, 2002). Laterites are ancient weathered profiles that are typically formed under tropical climates (Volkoff, 1998) and comprise five horizons underneath the topsoil: ferricrete, mottled zone, pallid zone, saprolite, and (parent) bedrock (Figure 1A). The ferricrete can be several meters thick, either occurring as a ferruginous crust (duricrust) with a pisolitic structure (Anand and Gilkes, 1987; Beauvais and Colin, 1993), or in a nodular form (ironstone gravel) (Tille et al., 2001). The mottled and pallid (bleached) zones comprise kaolinitic clay and quartz grains; while the mottled zone also contains iron oxides (e.g., goethite and haematite) and aluminum minerals (e.g., gibbsite) (Mulcahy, 1960). The saprolite (isovolumetric weathering product) layer may also contain some haematite, gibbsite and goethite (Girard et al., 1997; Théveniaut and Freyssinet, 1999).

Lateritic soils are infertile due to prolonged weathering of the bedrock. Nitrogen (N), phosphorus, and potassium are severely depleted, while some nutrients are inaccessible as they bind with lateritic compounds (Orians and Milewski, 2007). This extensive leaching also causes lateritic soils to be acidic, with *Eucalyptus* litter leachates also stimulating soil acidification and iron mobilization (Ellis, 1971; Bernhard-Reversat, 1999; Anand and Paine, 2002). Low soil organic matter (SOM) and clay contents in near-surface horizons result in a low cation exchange capacity (CEC), or even confer an anion exchange capacity that promotes further leaching (Wong and Wittwer, 2009). The pallid zone is usually highly saline owing to the accumulation of salts over thousands of years (Lewis, 1985; Brouwer and Fitzpatrick, 2002), making it inhospitable to many microbial and plant species. Since agricultural soils in WA are derived from eroded and redistributed lateritic material, they have inherited many of these limitations. This may have important implications for crop management in these areas, since microbial communities are critical in performing functions vital for soil health and crop growth, such as nutrient cycling and disease suppression (Frankenberger et al., 1989; Barea et al., 2005; Van Der Heijden et al., 2008).

Water repellence is a common feature of WA soils, primarily caused by hydrophobic waxes from the leaves of *Eucalyptus* and other plants adhering to soil particles, thereby reducing soil permeability and water infiltration (reviewed by Doerr et al., 2000; Walden et al., 2015). Microbial activity can gradually alleviate soil water repellence (SWR) by degrading these hydrophobic compounds (Ma’shum and Farmer, 1985; Roper, 2004). However, microbes may also exacerbate water repellency since they breakdown hydrophilic SOM content more rapidly, leading to the accumulation of hydrophobic compounds. Furthermore, microbial exudates can become hydrophobic under hot, dry conditions (Savage et al., 1969; Hallett and Young, 1999).

Soil microbial communities display significant and broad responses to geophysical attributes and vegetation. Soil pH is frequently reported as the strongest predictor of spatio-temporal variations in soil microbial communities (Lauber et al., 2009; Stone et al., 2015; Siles and Margesin, 2016; Zhang et al., 2017; Delgado-Baquerizo et al., 2018), with neutral soils typically exhibiting greater diversity relative to acidic soils (Fierer and Jackson, 2006; Rousk et al., 2010). Possibly due to its associations with soil pH and nutrient holding capacity (Liang et al., 2006), CEC has been shown to significantly affect soil microbial community functioning and composition (Pankhurst et al., 2001; Andrew et al., 2012; Docherty et al., 2015). Soil electrical conductivity (EC) – which is closely linked to salinity, CEC and SOM (Smith et al., 2002) – is reported to negatively correlate with bacterial diversity (Siles and Margesin, 2016). Other soil factors reported to co-vary with bacterial diversity include texture (silt, sand, and clay distribution) and total carbon content (Andrew et al., 2012), which are both related to the soil’s specific surface area (SSA) and CEC (Ersahin et al., 2006). Research on microbial community responses to soil water repellency is limited; although an increased dominance of Actinobacteria, including wax-degrading actinomycetes, have been associated with water repellent soils (Roper, 2004; Braun et al., 2010; Lozano et al., 2014). Vegetation cover can significantly alter microbial communities, but these responses are inconsistent and unpredictable owing to varying soil physicochemical attributes (Jangid et al., 2011; Lammel et al., 2015). Indeed, associations between vegetation and soil microbial communities range from being very weak or absent in some studies (Kielak et al., 2008; Štursová et al., 2016) to significant elsewhere (Shi et al., 2015; Stone et al., 2015; Yarwood et al., 2015).

In this study, we assess compositional and functional shifts in the soil bacterial community along a lateritic hillslope in WA. The study site represents an ideal location to examine biotic and abiotic dynamics in relation to laterite profiles since gradual erosion has resulted in the exposure of different weathering zones which has resulted in a geochemical gradient in a small localized area. Building on the work carried out by Gleeson et al. (2016), we conducted further investigations to elucidate how the soil bacterial community structure and function changes in response to the highly localized variations in edaphic variables (pH, EC, SWR, and soil texture) associated with different lateritic horizons and vegetation.

## Materials and Methods

### Study Site

This study was conducted in February 2015 at Fox Hill, located 120 km southeast of Perth at the University of Western Australia’s research farm Ridgefield, West Pingelly (116°59′31″ E, 32°30′23″ S) (Figure 1B). Fox Hill is one of five hill complexes in the Avon River CZ Observatory (AR-CZO) (Critical Zone Exploration Network, 2018). Another hill within the AR-CZO, Avery Hill, was the site of a study by Gleeson et al. (2016) who also investigated environmental influences on soil microbial communities along a lateritic profile. In this study, we used high-throughput sequencing to further characterize the bacterial communities of lateritic profiles using two contrasting transects along Fox Hill, coupled with soil physicochemical and vegetation cover measurements. The AR-CZO is part of the Eastern Darling Range, which exhibits a typical laterite deposit over granitic and doleritic bedrock (Anand and Gilkes, 1987). The climate is Mediterranean (hot, dry summers, and cool, wet winters), with an average annual rainfall of 425 mm and a mean temperature range from 10.4 to 23.4°C (Bureau of Meteorology, 2018). The laterite here is believed to have formed during the Cretaceous to mid-Miocene era (approx. 140 Ma) and has been well preserved as it has not been subjected to glaciation since the Late Carboniferous and Early Permian (Anand and Paine, 2002; Hopper and Gioia, 2004; Anand and de Broekert, 2005), although fluvial incision and lateral erosion has resulted in a dissected landscape (Mulcahy, 1960). Differing histories of hillslope lowering and evolution have resulted in varying exposures of the laterite profile along the hillslope (as designated by depth within the laterite profile). Normally these different horizons are buried beneath duricrust, hence this site provides a rare opportunity to access them and study their soil bacterial communities in relation to the CZ along this representative and ancient weathering profile that inputs, via colluviation, into highly vulnerable agricultural soils at its base.

The southeastern end of the hillslope was divided into eight transects (T35, T60, T90, T110, T140, T160, T185, and T210), named according to the corresponding compass bearing (with 0° north as reference). Soil samples were collected for physicochemical measurements [pH, EC and Molarity of Ethanol Droplet (MED)] at several points along each transect (Figure 1B and Supplementary Figure S1). T140 and T210 were chosen as principal transects for further detailed investigation, which involved additional physical (soil texture, water content, bulk density (BD), vegetation cover) and biological (16S rRNA gene sequencing) analyses. These principal transects were chosen because they represented contrasting aspects of the hill and varying depths of the lateritic weathering profile. Hillslope morphology was used to subdivide each transect into sampling sections (in pasture, bottom, mid, near top, top, and plateau). Surficial soil samples taken from the plateau of the hill represent the duricrust; the upper and mid sections of the slope correspond to the mottled and pallid zones; the bottom sections represent the base of the pallid zone comprising colluvial soil and the underlying saprolite layer (Gleeson et al., 2016) (Figure 1 and Supplementary Figure S1). In-pasture sections, collected for T210 only, were located in the paddock.

### Field Methods

Within each section of the 8 transects, two sampling points were chosen at 25% and 75% of the total section length (named S1 and S2, respectively, Supplementary Figure S1). Three replicate soil samples were collected from each sampling point, one on the mid-line of the transect and one either side (1 m perpendicular) of the transect. Soil samples for BD and gravimetric water content analyses were taken from the top 0–10 cm surface of each subsection on T140 and T210 (Grossman and Reinsch, 2002). Soil samples from each transect section were then pooled, homogenized (*n* = 6 per section), and cooled until laboratory analysis. Samples for DNA extraction (*n* = 63) were stored in-field at −20°C in dry-ice coolers and subsequently at −40°C upon arrival at the laboratory. Down-plot photographs were taken for ground cover percentage and vegetation type assessments via a line-point intercept method (Herrick et al., 2005) using ImageJ (v.2) software. Tree canopy cover was visually estimated, and ground cover type was recorded for both the most dominant (rank 1) and second-most dominant (rank 2) vegetation type. To further define CZ architecture and assess the depth to the pallid zone along hillslope position, we conducted a two-dimensional electrical resistivity tomography (ERT) survey using a long linear array of electrodes spaced 4 m apart (4point light, Lippman, Germany) across the transverse profile of the hill (396 m), encompassing transects T210 and T35 (northeast-facing transect). The apparent resistivity values were inverted using the program RES2DINV (Geotomo software) following Leopold et al. (2013) (Figure 1C).

### Laboratory Methods

Bulk density and loose bulk soil samples (unsieved) from the two main transects were weighed before and after oven-drying (105°C for 24 h) to determine dry BD and volumetric water contents (VWC). For all other analyses, soil samples were oven-dried (40°C) and sieved (2 mm). A total of 71 soil samples were used for separate measurements of MED, pH and EC, comprising 2 samples per section for each transect. Soil pH and EC were determined in a soil:solution ratio of 1:5 (v/v) in CaCl_2_ (0.01 M) for pH and distilled water for EC. Water repellency was assessed using the MED method (King, 1981). Soil texture analysis was conducted on 50 g of soil per subsection of T140 and T210 using the standard sodium hexametaphosphate dispersion and pipetting method (Gee and Or, 2002). The particle size class distribution was determined using the German size-particle classification system for sand (<2000-63 μm), silt (<63-2 μm) and clay (<2 μm).

### Statistical Analysis of Vegetation and Soil Biochemical Data

Statistical differences in vegetation cover between sections and transects were determined using chi-square (χ^2^) tests. One-way analysis of variance (ANOVA) tests were used to test for significant differences in soil properties (pH, EC, SWR, VWC, and soil texture) between and within transects, and Tukey’s honestly significant difference (HSD) tests were performed subsequently as a *post hoc* test. VWC and EC values were log-transformed to attain normal distributions (Shapiro Wilks test *P* > 0.05). Non-parametric tests (Kruskall-Wallis and *post hoc* Dunn’s test) were used to analyse MED results as they did not follow a normal distribution even when transformed. Plateau and in-pasture samples were removed when testing for difference between all eight transects, since they were not taken from all transects.

### DNA Extraction and 16S rRNA Gene Sequencing

DNA was extracted from a total of 63 soil samples using the PowerSoil^®^ DNA Isolation Kit (MoBio, Carlsbad, CA, United States) following manufacturer instructions. The DNA concentration of each sample was quantified using a fluorescence approach (Qubit^®^ 2.0 Fluorometer, Life Technologies, Mulgrave, Victoria, Australia), before dilution to 1 ng μL^–1^ prior to PCR amplification. The V4–V5 region of the universal prokaryotic 16S rRNA gene was amplified using primers 515F and 806R (Caporaso et al., 2010b) modified according to Whiteley et al. (2012) to enable multiplexing of samples (Hamady et al., 2010). PCR was conducted according to Gleeson et al. (2016). After gel electrophoresis (1.5%, 80 V for 40 min), DNA bands were cut and cleaned using the Wizard^®^ SV Gel and PCR Clean-Up System (Promega, Alexandria, NSW, Australia), before pooling the barcoded products and purifying the DNA using Agencourt AMPure XP following the manufacturer instructions (Beckman Coulter, Brea, CA, United States). High-throughput amplicon sequencing was performed using the Ion Torrent Personal Genome Machine (Life Technologies Australia Pty Ltd., Mulgrave, VIC, Australia) (Rothberg et al., 2011) and 400 base pair chemistry.

### Sequence Data Processing

Sequences were processed and analyzed using MacQIIME (Caporaso et al., 2010a). Multiplexed sequences were split into individual samples and barcodes were removed using Cutadapt v.1.12 (Martin, 2011). Sequences with a minimum quality score <20 and those identified as chimeras were removed using USEARCH (Edgar, 2010). Sequences were clustered into OTUs based on 97% sequence similarity and taxonomy was assigned using the Greengenes database version 13.5 (McDonald et al., 2012). Singletons and OTUs classified as mitochondria and chloroplasts were removed before rarefaction to 2,158 sequences per sample, which was the minimum count per sample after removing OTUs with a frequency <0.005% (Gihring et al., 2012). During filtering, five samples were removed due to insufficient reads or quality, resulting in 58 soil samples for analyses.

Trimmed sequence reads of all samples were deposited in the Sequence Read Archive (SRA) of the National Centre for Biotechnology Information (NCBI) under Bioproject accession number PRJNA494632^1^.

### Bacterial Diversity and Statistical Analysis

Alpha (α)-diversity measures (observed species (OTUs), Chao1 richness, Faith’s phylogenetic diversity (PD), Shannon’s Index) were calculated for samples grouped by location using QIIME and statistical differences were checked using a non-parametric two-sample *t*-test (Monte Carlo). Beta (β)-diversity metrics were calculated using unweighted UniFrac measures and Bray-Curtis dissimilarity matrix.

β-diversity (unweighted UniFrac distances) was visualized using both unconstrained (PCoA) and constrained (dbRDA) (Shankar et al., 2017) ordination methods, as recommended by Anderson and Willis (2003). These ordination plots were created using the *phyloseq*, *vegan* and *ggplot2* packages in R version 3.5.1 (R Core Team, 2017). Environmental vectors depicting the contribution of the different edaphic variables toward bacterial community diversity were added to the dbRDA plot using the *envfit* function in *vegan* (Oksanen et al., 2018), the statistical significance of which was tested using ANOVA. PERMANOVA (Anderson, 2001) tests were conducted using the *adonis* function in R with 9,999 permutations to determine the amount of variation in average community composition (unweighted UniFrac matrices) that could be explained by each of the measured variables. The permutations were restricted to within the sampling location (transect and section) using the “strata” option in the *adonis* function. Pairwise comparisons of β-diversity between samples grouped by location were tested using the function *calc_pairwise_permanovas* in the MCTOOLSR package (Leff, 2016), which implements the *adonis* function and corrects *P-*values for multiple testing with false discovery rate (FDR) corrections. PERMDISP tests were conducted using the *betadisper* function to check for differences in group dispersions, equating to unequal structural variability of bacterial communities among groups (Erwin et al., 2012). When significant PERMDISP results indicated homogeneity was not satisfied, Tukey pairwise comparison tests were performed to determine which groups had significantly different dispersions. For these tests, continuous variables were ranked to create groups for comparison (Supplementary Table S1) and Benjamini–Hochberg FDR applied to correct for multiple testing (Benjamini and Hochberg, 1995). A heatmap displaying the relative abundance of the most dominant phyla (minimum abundance of 1% in at least one sample) was created using the *ampvis2* package in R.

Statistical differences in the relative abundances of OTUs between sampling groups were calculated by performing ANOVA tests with Benjamini–Hochberg FDR (filtered to a minimum effect size of 0.7 and *q* < 0.05) using the software Statistical Analysis of Metagenomic Profiles (STAMP) v. 2.1.3 (Parks et al., 2014). Significant results were further investigated using a Tukey–Kramer *post hoc* test (minimum effect size 0.7, *P* < 0.05).

The functional composition of the soil microbial communities was predicted using PICRUSt (Phylogenetic Investigation of Communities by Reconstruction of Unobserved States), following the documentation^2^ using default settings (Langille et al., 2013). KEGG pathways that differed significantly (*q*-value < 0.01) between sample groups were identified in STAMP using the Tukey–Kramer *post hoc* test with Benjamini–Hochberg FDR and a minimum effect size of 0.75.

## Results

### Vegetation Coverage

The dominant vegetation cover at the site was Salmon gum (*Eucalyptus salmonophloia*) and Ghost gum (*Corymbia papuana*^3^). Ground vegetation cover was slightly higher on T210 (mean 59.5%) than T140 (mean 51.3%), but this result was not significant (ANOVA = 3.34, *P* = 0.086) (Supplementary Table S2). T140 vegetation was dominated by *Eucalyptus*, whilst on T210 it shifted from a mixture of spring oats and *Eucalyptus* in the plateau and upper sections, to wheat and native grasses in the lower slope sections (Supplementary Table S2).

### Soil Bulk Density, Texture, and Water Content

The BD of the topsoil (0–10 cm) along the two principal transects (T140 and T210) ranged from 0.36 to 1.09 g cm^–3^ which is similar to that reported for vegetated lateritic soils elsewhere (Sherman et al., 1953). Dry weather conditions prior to and during sampling contributed to the low soil VWC which averaged 0.05 cm^3^ water cm^–3^ soil, indicating a water-stressed environment.

The surface soil texture was sandy loam (U.S. Department of Agriculture, 1993), containing on average 72.5% sand, 12% silt, and 15.5% clay-sized particles. Soil texture composition varied significantly between layers of the weathering profile (sections), but not between transects. Clay content on both of the principal transects was higher in the upper sections (top and near top, corresponding to the mottled zone) than on the plateau (*P* < 0.03) (Table 1). T210 exhibited greater variation in soil texture, with significantly higher proportions of silt in the plateau samples and sand in the lower (bottom and in-pasture) sections (Table 1).

**TABLE 1:** Topsoil characteristic measurements at transect sections (means).

**Sampling point**	**pH**	**MED (mol L^–1^)**	**EC (μS cm^–1^) ^‡^**	**Clay (%)**	**Silt (%)**	**Sand (%)**	**VWC (cm^3^ cm^–3^)**	**BD (g cm^–3^)**
**T140**								
Bottom	$4.58{}^{\mathrm{ab}}$	$1.20{}^{a}$	$139.50{}^{a}$	$13.69{}^{\mathrm{ab}}$	$11.41{}^{a}$	$74.91{}^{a}$	$0.03{}^{b}$	$1.05{}^{a}$
Mid	$4.16{}^{\mathrm{bc}}$	$1.60{}^{a}$	$1781.00{}^{a}$	$14.84{}^{\mathrm{ab}}$	$11.37{}^{a}$	$73.57{}^{a}$	$0.05{}^{\mathrm{ab}}$	$1.02{}^{a}$
Near Top	$3.65{}^{\mathrm{cd}}$	$1.34{}^{a}$	$2681.00{}^{a}$	$16.13{}^{a}$	$11.56{}^{a}$	$72.21{}^{a}$	$0.08{}^{a}$	$1.09{}^{a}$
Top	$3.57{}^{d}$	$1.34{}^{a}$	$2092.50{}^{a}$	$17.95{}^{a}$	$14.22{}^{a}$	$67.30{}^{a}$	$0.07{}^{\mathrm{ab}}$	$1.03{}^{a}$
Plateau	$5.08{}^{a}$	$2.40{}^{a}$	$149.00{}^{a}$	$9.29{}^{b}$	$12.39{}^{a}$	$78.32{}^{a}$	$0.04{}^{\mathrm{ab}}$	$0.93{}^{a}$
**T210**								
In-Pasture	$4.75{}^{a}$	$1.14{}^{\mathrm{bc}}$	$146.85{}^{a}$	$12.10{}^{\mathrm{cd}}$	$11.48{}^{\mathrm{bc}}$	$76.42{}^{a}$	$0.03{}^{a}$	$1.03{}^{a}$
Bottom	$4.84{}^{a}$	$0.07{}^{c}$	$67.15{}^{a}$	$8.54{}^{d}$	$10.24{}^{\mathrm{bc}}$	$81.23{}^{a}$	$0.04{}^{a}$	$1.09{}^{a}$
Mid	$4.54{}^{\mathrm{ab}}$	$2.54{}^{\mathrm{ab}}$	$253.10{}^{a}$	$16.17{}^{\mathrm{bc}}$	$12.73{}^{b}$	$71.11{}^{b}$	$0.03{}^{a}$	$0.81{}^{a}$
Near Top	$4.09{}^{\mathrm{bc}}$	$3.07{}^{a}$	$202.95{}^{a}$	$22.95{}^{\mathrm{ab}}$	$11.01{}^{\mathrm{bc}}$	$66.05{}^{b}$	$0.02{}^{a}$	$0.36{}^{a}$
Top	$3.86{}^{c}$	$2.40{}^{\mathrm{ab}}$	$123.50{}^{a}$	$24.16{}^{a}$	$9.40{}^{c}$	$66.45{}^{b}$	$0.03{}^{a}$	$0.74{}^{a}$
Plateau	$4.93{}^{a}$	$2.40{}^{\mathrm{ab}}$	$100.85{}^{a}$	$14.63{}^{\mathrm{cd}}$	$15.68{}^{a}$	$69.69{}^{b}$	n/a	n/a

*Letters indicate significantly different values assessed between sections within each individual transects (Tukey’s HSD test *P* < 0.05). **^‡^**T210 data was log-transformed before performing statistical tests (raw data shown); MED, molarity of ethanol droplet; EC, electrical conductivity; VWC, volumetric water capacity; BD, bulk density.*

### Soil pH

Overall, the hillslope soil pH was acidic (Supplementary Figure S2A), with a clear band of lower soil pH across the upper sections of the slope correlating with visible surface erosion and outcropping of characteristically sub-surface material in this area. When pooling data from all 8 transects, the bottom sections had a significantly higher pH than the top (*P* = 0.008) and near top (*P* = 0.038) sections. Similarly, the pH of the in-pasture samples was significantly higher than the top and near top sections (*P* = 0.004 and *P* = 0.001, respectively). However, when comparing the two principal transects in isolation from the additional transects, there was no significant difference in pH (*P* = 0.085).

On both of the principal transects, soil pH was significantly lower in the top sections in comparison to the plateau (T140 *P* = 0.002; T210 *P* = 0.002), mid (T140 *P* = 0.027; T210 *P* = 0.016) and bottom (T140 *P* = 0.003; T210 *P* = 0.002) sections (Table 1). Likewise, both transects also had a significantly lower soil pH in the near top sections relative to the plateau (T140 *P* = 0.002; T210 *P* = 0.005) and bottom (T140 *P* = 0.006; T210 *P* = 0.01). The pH of the mid-section of T140 was significantly lower than the plateau (*P* = 0.009).

### Soil EC

When comparing the main transects, EC was significantly higher on the T140 slope (mean 1504 μS cm^–1^) compared to T210 (mean 149 μS cm^–1^) (Supplementary Figure S2B, *P* = 0.005), indicating greater soil salinity on the southeastern slope (T140).

There were no significant within-slope differences in EC on either transect, although the mid to top sections generally had higher EC values, which correspond to the sections closest to the pallid zone before it is covered by colluvium further down the slope. The highest EC measurements (>4,000 μS cm^–1^) occurred along the upper slope sections of Fox Hill on its north- and south-eastern aspects (T60, T110, T140, and T160), which coincided with the outcropping of high-salinity pallid zone deposits.

### Soil Water Repellency

Focussing on the two principal transects, the only significant within-transect differences in the mean MED results occurred on T210 where the bottom section MED value was significantly lower than those of the mid (*P* = 0.007), near top (*P* = 0.002), top (*P* = 0.009) and plateau (*P* = 0.009) sections (Table 1 and Supplementary Figure S2C).

### Soil Abiotic Factor Correlations

We found correlations between several soil characteristics. Clay content was positively correlated with gravimetric water content (*R* = 0.67, *P* = 0.01) and SWR was negatively correlated with BD (ρ = 0.77, *P* = 0.002). Soil pH correlated negatively with VWC (ρ = 0.82, *P* = 0.001) and positively with secondary ground cover vegetation, soil pH being lowest where vegetation cover was dominated by *Eucalyptus* (*R* = 0.64, *P* = 0.004).

### Microbial Community Composition

**FIGURE 2:**
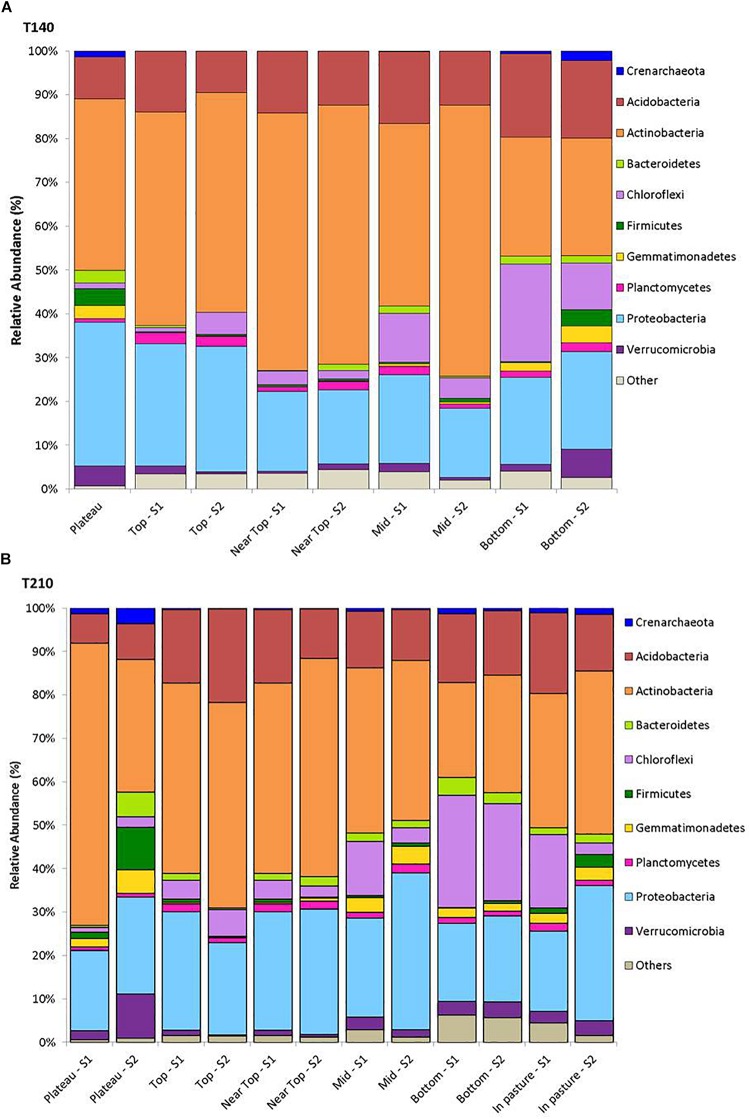
Mean relative abundance of the dominant phyla (>1%) for samples from each sampling location on T140 **(A)** and T210 **(B)**.

After filtering, a total of 217,396 sequences were obtained from all 58 soil samples from the two main transects, comprising a total of 24,104 operational taxonomic units (OTUs). The rarefied data set comprised a total of 2,849 OTUs assigned to 19 phyla, 47 classes, 73 orders, 90 families, and 87 genera of archaea and bacteria. The 10 most abundant phyla across all samples were Actinobacteria (41.6%), Proteobacteria (23.0%), Acidobacteria (14.0%), Chloroflexi (8.5%), Verrucomicrobia (2.6%), Gemmatimonadetes (1.8%), Bacteroidetes (1.8%), Planctomycetes (1.5%), Firmicutes (1.5%) and Thaumarchaeota (0.8%); with 1.8% of reads being unassigned to any phylum (Figure 2).

### Bacterial Community Diversity

Although α-diversity measures exhibited no significant differences between groups, the upper sections of both transects consistently had the lowest diversity according to all tested metrics. The bottom and in-pasture sections generally had the highest *α*-diversity (Supplementary Table S3).

**FIGURE 3:**
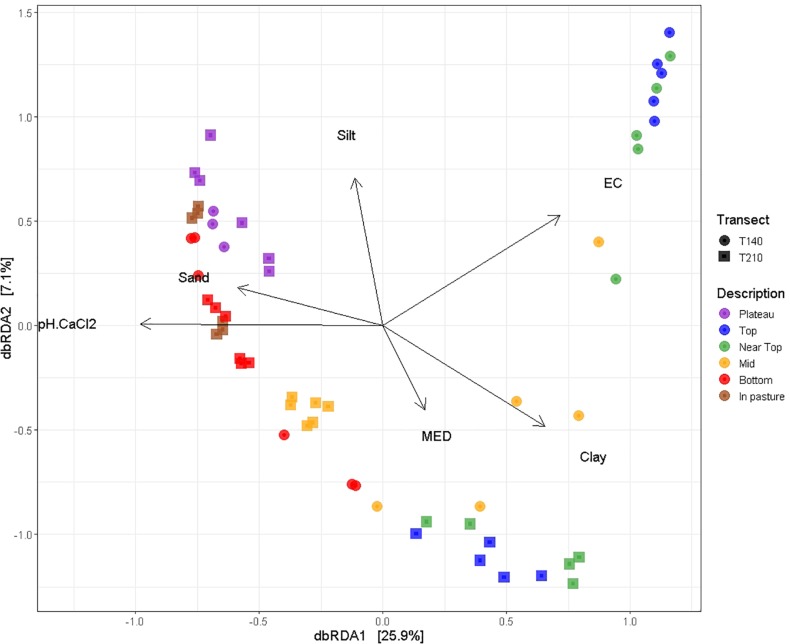
dbRDA ordination plot using unweighted UniFrac distance matrices with environmental variables (pH, EC, MED, and soil texture) mapped on using the *envfit* function (*vegan* package in R). Both the first (*P* = 0.001) and second (*P* = 0.001) dbRDA axes significantly explained the variation in the bacterial community composition.

Permutational multivariate analysis of variance (PERMANOVA) tests indicated that all tested variables significantly affected soil bacterial community composition (OTU presence/absence), with soil EC (ranked) (*R*^2^ = 0.19, *P* = 0.0001), and soil pH (ranked) (*R*^2^ = 0.31, *P* = 0.0001) having the strongest correlations (Table 2). SWR (MED ranked) (*R*^2^ = 0.07, *P* = 0.03) and clay % (*R*^2^ = 0.02, *P* = 0.005) exhibited weaker yet significant associations with microbial β-diversity. Pairwise PERMANOVA indicated that the strongest contrasts in β-diversity occurred between samples of the upper (top and near top) sections and those of the plateau and lower (bottom and in-pasture) sections on both transects (*P* ≤ 0.02, *R^2^* > 0.4; Supplementary Table S4). However, permutational multivariate analysis of dispersion (PERMDISP) tests also yielded significant results, indicating that the PERMANOVA results may be partially caused by differences in the group dispersions, rather than their centroids (Table 3 and Supplementary Table S5). Nonetheless, the PERMANOVA results were supported by both the principal coordinate analysis (PCoA) and the distance-based redundancy analysis (dbRDA) which indicated a distinct clustering of the upper section communities away from those of the plateau and lower sections, particularly on T140 (Figure 3 and Supplementary Figure S3). The dbRDA plot also suggested that the upper sections exhibited the greatest contrast between transects, as they formed two separate clusters. The dbRDA environmental vectors indicated that these upper section microbial communities were most strongly associated with soil EC on T140 (*P* = 0.001), and MED (*P* = 0.001) and clay content (*P* = 0.027) on T210. The in-pasture, bottom and plateau communities of both transects correlated strongly with soil pH (*P* = 0.001) and sand content (*P* = 0.011).

**TABLE 2:** PERMANOVA (using the function *adonis*) results using unweighted UniFrac (9999 permutations).

**Factor**	**Df**	**Sum of squares**	**Mean squares**	***F*-value**	***R^2^***	***P*-value**
pH_CaCl2_^†^	3	3.573	1.191	11.243	0.306	0.0001^∗∗∗^
EC^†^	7	2.217	0.317	2.990	0.190	0.0001^∗∗∗^
MED^†^	3	0.793	0.264	2.494	0.068	0.0323^*^
Silt	1	0.263	0.263	2.480	0.022	0.7578
Sand	1	0.266	0.266	2.510	0.023	0.4242
Clay	1	0.231	0.231	2.180	0.020	0.0054^∗∗^

^†^ranked data; significance levels: ^∗∗∗^*P* < 0.001, ^∗∗^*P* < 0.01, ^*^P < 0.05.

**TABLE 3:** PERMDISP test for homogeneity results using unweighted UniFrac.

**Parameter**	**Df**	**Sum of squares**	**Mean squares**	***F*-value**	***P*-value**
Sampling location (Transect and section)	10	0.052559	0.0052559	5.1932	<0.0001^∗∗∗^
pH_CaCl2_${}^{†}$	3	0.0405	0.0135	6.3757	0.0009^∗∗∗^
MED${}^{†}$	3	0.1381	0.0138	7.6943	0.0002^∗∗∗^
EC${}^{†}$	7	0.0680	0.0097	4.4453	0.0007^∗∗∗^
Clay	19	0.0685	0.0036	4.5889	<0.0001^∗∗∗^
Silt	19	0.0685	0.0036	4.5889	<0.0001^∗∗∗^
Sand	18	0.1329	0.0738	3.9581	0.0002^∗∗∗^

^†^ranked data; significance levels: ^∗∗∗^*P* < 0.001, ^∗∗^*P* < 0.01, ^*^P < 0.05.

### Differentially Abundant Taxa Within-Transects

**FIGURE 4:**
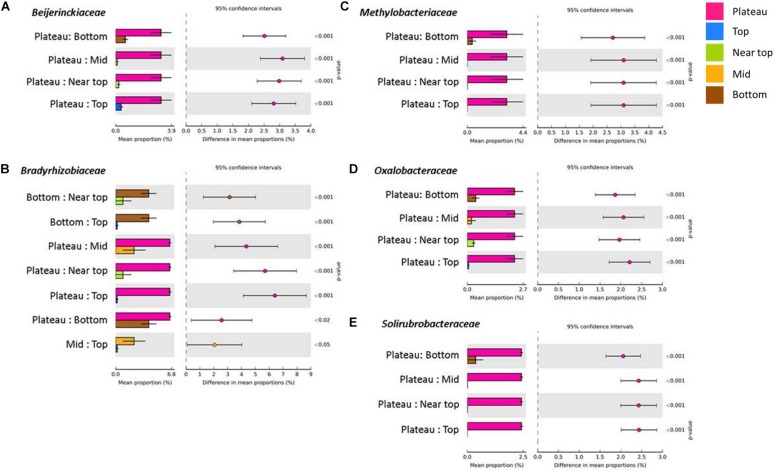
Families within the phyla Proteobacteria [**(A)** *Beijerinckiaceae*, **(B)** *Bradyrhizobiaceae*, **(C)** *Methylobacteriaceae* and **(D)** *Oxalobacteraceae*] and Actinobacteria [**(E)** *Solirubrobacteraceae*] with significantly higher mean proportions in the plateau sections of T140 (Benjamini–Hochberg FDR *P* < 0.05, difference in mean proportion > 1.5%).

**FIGURE 5:**
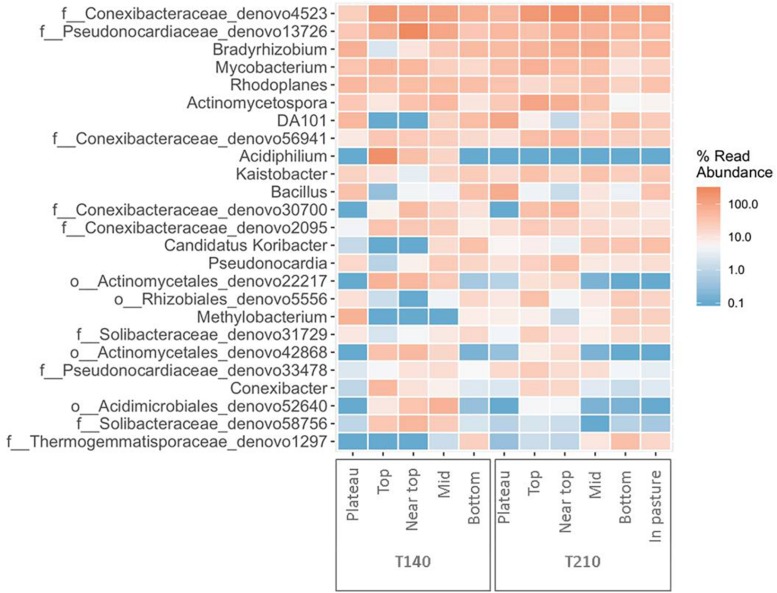
Heatmap of the top 25 most abundant genera in each section of the two principal transects (created using the package *ampvis2* in R).

On T140, the mean relative abundance of Gemmatimonadetes was significantly higher in the plateau and bottom sections relative to all others (*P* < 0.0001). The only differentially abundant phylum on T210 was Chloroflexi, which was significantly more numerous in the bottom section (*P* < 0.0001). When examining within-transect differences at a higher taxonomic resolution, several members of the Actinobacteria were more prevalent in the T140 plateau relative to the other sections. These included the families *Kineosporiaceae*, *Micromonosporaceae*, and *Solirubrobacteraceae* (all *P* < 0.0001); genera *Kibdelosporangium* (*P* < 0.0001), *Dactylosporangium* (*P* < 0.0001), *Modestobacter* (*P* < 0.0001), *Rubrobacter* (*P* < 0.001), and *Amycolatopsis* (*P* < 0.0001) (Figure 4 and Supplementary Figure S4). Other taxa with increased abundance in the T140 plateau belonged to the phyla Proteobacteria [families *Beijerinckiaceae*, *Bradyrhizobiaceae*, *Comamonadaceae*, *Methylobacteriaceae*, and *Oxalobacteraceae*; genera *Methylibium*, *Ramlibacter*, *Roseomonas*, and *Methylobacterium* (all *P* < 0.0001)]; Bacteroidetes [genera *Pedobacter* (*P* = 0.0004) and *Segetibacter* (*P* = 0.0002)] and Firmicutes [genera *Cohnella* (*P* = 0.0001), *Alicyclobacillus* (*P* = 0.0002), and *Ammoniphilus* (*P* = 0.0003)] (Figures 4, 5, and Supplementary Figure S4).

**FIGURE 6:**
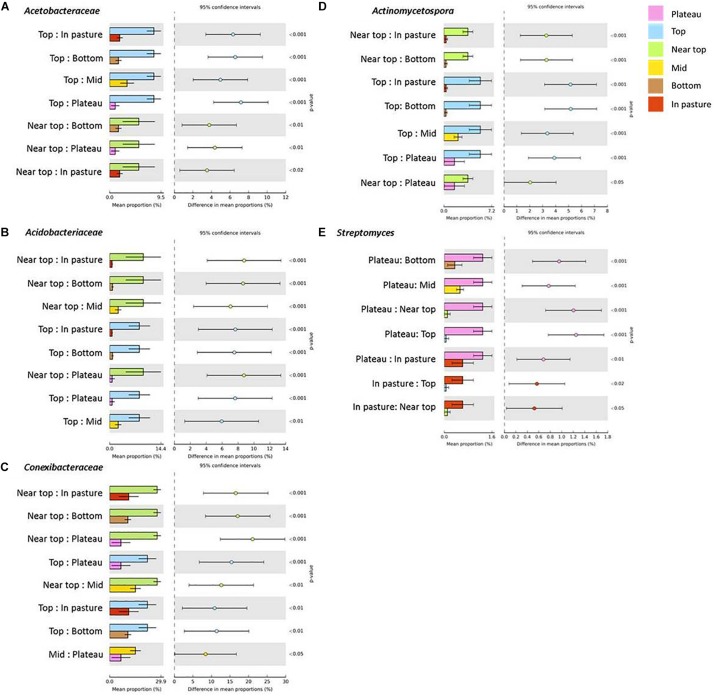
Bacterial taxa with significantly higher mean abundances in the upper (near top and top, **A–D**) and plateau **(E)** sections of T210 relative to all other sections of this transect (Benjamini–Hochberg FDR *P* < 0.05, difference in mean proportion > 2%).

The upper sections of both transects exhibited higher abundances of *Acetobacteraceae*, *Acidobacteriaceae*, and *Conexibacter* relative to all other sections (Figure 6 and Supplementary Figure S4). On T140, the top section had significantly more *Acidiphilium* OTUs (*P* = 0.0001, Figure 5); the near top was enriched in an unidentified member of the family *Pseudonocardiaceae denovo13726*; while *Koribacteraceae* (*P* < 0.0001) and *Cryocola* (*P* < 0.0001) were more numerous in the bottom section (Supplementary Figure S4). *Actinomycetospora* (*P* < 0.0001) and *Streptomyces* (*P* < 0.0001) were significantly more abundant in the upper and plateau sections of T210, respectively (Figures 5 and 6).

### Between-Transect Differences in Bacterial Taxa Abundances

**FIGURE 7:**
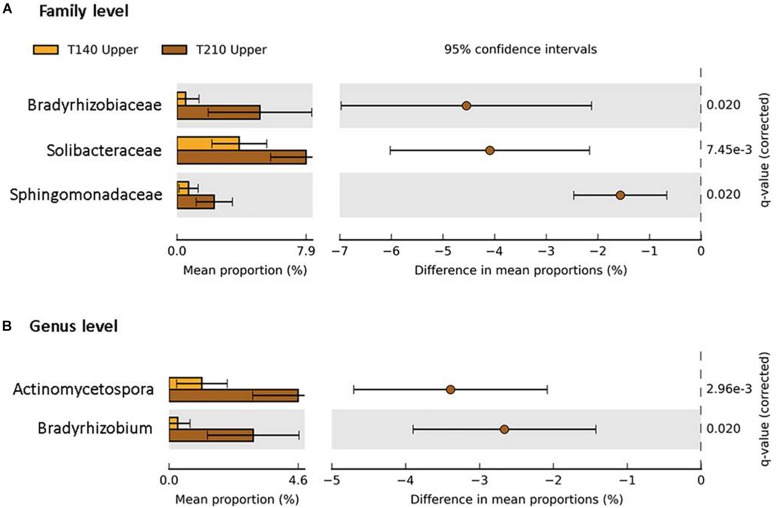
Taxa with significantly different abundances between the upper sections of the two main transects T140 (orange bars) and T210 (brown bars) at the family **(A)** and genus **(B)** level (Welch’s *t*-test *P* < 0.05 (Benjamini–Hochberg FDR), difference in mean proportion > 1.5%).

When comparing the two transects, significant differences in taxa abundances were limited to the upper sections. Relative to those of T140, the T210 upper sections were enriched in members of the phyla Proteobacteria (families *Bradyrhizobiaceae* and *Sphingomonadaceae*; genus *Bradyrhizobium*), Actinobacteria (genus *Actinomycetospora*), and Acidobacteria (family *Solibacteraceae*) (*P* < 0.05; difference between mean proportions >1.5) (Figure 7).

### Soil Biotic Correlations

*Amycolatopsis* (phylum Actinobacteria) was the only OTU found to vary significantly with pH (ranked), being more numerous in the least acidic soils (pH 5.0–5.5) relative to all others (pH < 5). The most water repellent soils (MED 3–3.9 mol L^–1^) had significantly more *Salinispora* OTUs (*P* < 0.001), members of which grow optimally in saline environments, such as seawater (Ng et al., 2014).

### Functional Comparisons of Microbial Communities

**FIGURE 8:**
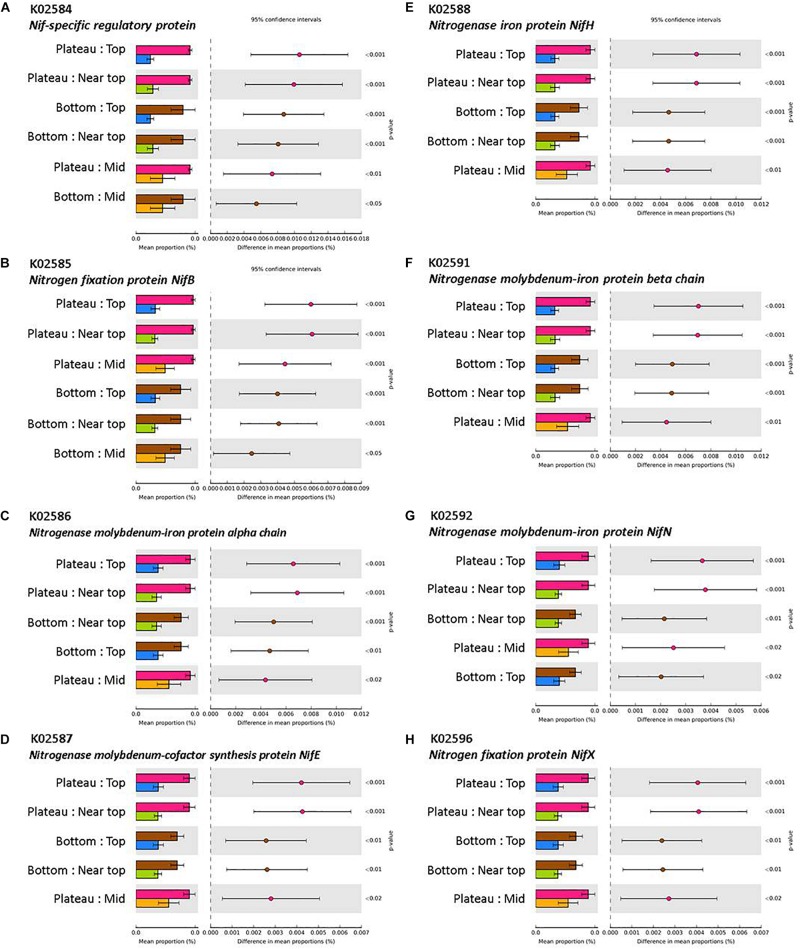
KEGG orthologs (KOs) involved in N-fixation via **(A)** Nif-specific regulatory protein, **(B,H)** Nitrogen fixation protein NifB and NifX, **(C,F,G)** Nitrogenase molybdenum-iron protein alpha chain, beta chain, and NifN, **(D)** Nitrogenase molybdenum-cofactor synthesis protein NifE, **(E)** Nitrogenase iron protein NifH, with significantly higher abundances in the plateau and bottom sections relative to all others (top, near top, and mid) on T140. KO abundances were calculated using PICRUSt and visualized in STAMP (minimum effect size = 0.7, Benjamini–Hochberg multiple test-corrected *q*-value < 0.05).

The mean nearest sequenced taxon index (NSTI) value was 0.11 (±0.003), indicating a good strength of reliability in the PICRUSt metagenome predictions. PICRUSt analysis indicated a significant effect of the weathering profile on the predicted functional traits of soil communities, with several pathways involved in metabolism significantly differing in abundance between the bottom and upper sections of both transects (*P* < 0.001; Table 4). KEGG orthologs (KOs) related to N-fixation were significantly more abundant in the plateau and bottom sections in comparison to all others on T140 (Figure 8).

**TABLE 4:** KEGG pathways predicted that differed significantly between sections of T140 and T210.

**Gene category**	**T140 (%)**					**T210 (%)**					
	**Plateau**	**Top**	**Near Top**	**Mid**	**Bottom**	**Plateau**	**Top**	**Near Top**	**Mid**	**Bottom**	**In-Pasture**
**Metabolism**											
Arginine and proline metabolism	1.28 cde	**1.36 a**	**1.33 ab**	1.30 bcd	1.26 e	**1.31 bc**	1.30 bcde	**1.32 abc**	1.28 cde	1.27 de	1.29 cde
Biotin metabolism	0.13 bcde	**0.14 a**	**0.14 ab**	0.13 cde	0.11 e	**0.14 abc**	0.13 cde	**0.13 bcd**	0.13 cde	0.11 e	0.12 de
Biosynthesis of unsaturated fatty acids	0.36 bcd	**0.40 a**	**0.41 a**	0.38 abc	0.35 d	**0.39 ab**	**0.39 ab**	**0.40 a**	0.37 bcd	0.36 d	0.36 cd
Tropane, piperidine and pyridine alkaloid biosynthesis	0.13 cd	**0.15 a**	**0.15 a**	0.14 cd	0.13 d	0.13 cd	**0.15 ab**	**0.15 ab**	0.14 bc	0.13 cd	0.13 cd
Fructose and mannose metabolism	0.55 cde	0.55 de	0.54 e	0.56 bcd	**0.57 a**	0.55 cde	**0.57 ab**	0.56 bcde	0.56 bcd	**0.58 a**	0.56 abc
Alanine, aspartate and glutamate metabolism	0.89 abcd	0.86 cde	0.85 de	0.88 bcde	**0.92 a**	0.90 abc	0.86 de	0.84 e	0.87 bcde	**0.91 ab**	**0.91 ab**
beta-Lactam resistance	0.05 abcd	0.04 e	0.04 de	0.05 bcd	**0.06 a**	0.04 de	0.05 cd	0.04 de	0.05 cd	**0.06 ab**	0.06 abc
Lipid biosynthesis proteins	0.97 abc	1.05 cd	1.12 d	1.03 cd	**0.93 a**	1.00 abc	1.02 bc	1.04 cd	0.98 abc	**0.93 a**	0.94 ab
Penicillin and cephalosporin biosynthesis	0.08 abcd	0.06 e	0.07 de	0.08 bcd	**0.10 a**	0.07 de	0.08 cd	0.07 d	0.08 cd	**0.09 ab**	0.09 abc
Nucleotide metabolism [Unclassified]	**0.02 ab**	0.01 f	0.01 def	0.01 cde	**0.02 ab**	**0.02 a**	0.01 ef	0.01 ef	0.01 bcd	**0.01 ab**	**0.01 abc**
Ubiquinone and other terpenoid-quinone synthesis	**0.28 ab**	0.26 cd	0.25 d	0.27 bcd	**0.28 ab**	**0.28 a**	0.26 cd	0.26 d	0.28 abc	**0.29 a**	**0.28 ab**
Biosynthesis of ansamycins	**0.06 a**	0.04 d	0.05 ab	0.06 ab	**0.06 a**	0.05 ab	0.05 cd	0.05 bc	0.05 abc	**0.06 a**	0.05 abc
Protein kinases	0.39 cd	0.40 cd	0.38 d	0.39 cd	**0.41 ab**	0.38 d	0.40 bcd	0.38 d	0.40 cd	**0.42 a**	0.41 abc
**Genetic information processing**											
Protein processing in endoplasmic reticulum	0.07 abc	0.06 de	0.06 cde	0.06 bcde	0.07 abc	**0.08 a**	0.06 e	0.06 e	0.07 abc	**0.07 ab**	0.07 abcd
**Cellular processes and signaling**											
Inorganic ion transport and metabolism [Unclassified]	0.34 bc	0.32 d	0.33 cd	0.34 bc	**0.36 a**	0.33 cd	0.33 cd	0.33 cd	0.35 abc	**0.36 ab**	**0.36 ab**

*Significantly higher frequencies are highlighted in bold (*P* < 0.001, effect size > 0.7). Letters indicate significantly different values assessed between sections of both transects (Tukey’s HSD test *P* < 0.05).*

Fewer differences were detected when grouping samples by pH, although those with a pH > 4 had more KOs for nitrous-oxide reductase and nitrous oxidase accessory protein in comparison to more acidic soils (pH < 4) (*P* < 0.001, Supplementary Figure S5). Soils with higher EC (>1200 μS cm^–1^) were enriched in KOs assigned as prephenate dehydrogenase and putative glutamine amidotransferase, in comparison to all other samples (*P* < 0.01). There were no apparent significant effects of MED (ranked) on predicted functions.

## Discussion

This study demonstrates that a suite of edaphic factors shaped by both weathering history and vegetation result in greatly divergent soil microbial communities along this lateritic profile. Multivariate analysis identified soil pH and EC as the strongest predictors of soil bacterial diversity, as reported in other studies (Meola et al., 2014; Kim et al., 2016; Tytgat et al., 2016). Sampling location (transect and section) also accounted for considerable variation in bacterial β-diversity, with plateau and lower sections exhibiting the most similar communities on both transects. Since these sections correspond to different layers of a well-preserved ancient weathering profile (Figure 1 and Supplementary Figure S1C), our findings support a link between mineralogy and soil microbial communities. This association has been detected across many environments (Carson et al., 2009; Jones and Bennett, 2014; Kim et al., 2015) including alpine glacier forefields in Switzerland (Meola et al., 2014) and naturally metal-rich soils in Australia (Reith et al., 2015). It is important to note that the bottom sections bordered an agricultural field, which may have affected these results since soil microbial communities are significantly affected by agricultural practices such as fertilizer inputs (Nguyen et al., 2018; O’Brien et al., 2018) and tilling (Babin et al., 2018).

Of all the abiotic soil variables measured, only pH and clay content varied significantly between sections on both transects. Soil pH was highest in the plateau sections, while the upper sections were the most acidic and had the highest clay content relative to all other sections. The lower pH of the upper sections is likely due to them corresponding with the mottled zone, which typically has low pH and high EC (McArthur, 1991; Tille et al., 2001). These characteristics may be explained by the high content of kaolinitic clay (positive correlation with soil EC) and salts (negative correlation with pH) in the mottled zone, in addition to the widely reported negative correlation between soil EC and pH, as supported by our results (Wang et al., 2008; Aini et al., 2014). On T210, it may also result from the dominance of eucalypt species in these sections as their leaf litter leachates promote cation leaching and acidification (Swift et al., 1979; Bernhard-Reversat, 1999) (Supplementary Table S2). Another factor affecting soil pH may have been the position on the slope, as soil pH has been shown to be lower at higher elevations possibly due to the downward transport of sediments and leaching of basic cations from upper hillslope sections (Smith et al., 2002; Hattar et al., 2010). Soil EC has also been reported to correlate with elevation, decreasing at lower elevations due to higher moisture accumulation (Smith et al., 2002; Hattar et al., 2010).

Another topographical measure that may have influenced soil physicochemical traits at our study site was slope gradient. Slope inclination can influence soil moisture content, as steeper slopes have greater runoff and transportation of sediments downslope (Hall, 1983). This runoff has been shown to result in a higher concentration of exchangeable bases (e.g., Ca^2+^, K^+^ and Na^+^) and, as a possible consequence, higher soil pH at the foot of the slope (Tsui et al., 2004). However, there are contrasting reports of the relationships between soil physicochemical attributes and slope features. For instance, Ribolzi et al. (2011) reported that steeper slopes in northern Laos had less runoff which they partially attribute to the lower infiltration rates observed on steeper slopes, while Hattar et al. (2010) found that neither soil pH or EC bore a significant correlation with slope steepness.

Soil water repellence varied significantly on T210, with the bottom sections being the least water repellent, possibly due to the accumulation of colluvial material at the bottom of the hillslope. The mean MED for plateau samples (both transects) and T210 mid and upper sections represented moderate to severe water repellency according to Jackson and Linskens (1991). On T140, this may be attributed to the low clay content which is characteristic of water repellent soils (Harper et al., 2000), with clay additions serving as an effective method to ameliorate SWR (Hall et al., 2010; Shanmugam et al., 2014). Another possible contributing factor is the occurrence of forest fires – which are frequent in WA – as these can result in increased soil hydrophobicity (DeBano, 2000). Furthermore, a positive feedback loop can establish as hydrophobic soils commonly form preferential flow paths and soil aggregates which may intensify drought events and inhibit microbial degradation of organic matter (Goebel et al., 2011).

The lower soil BD on T210 relative to T140 was attributed to the greater vegetation cover and clay content on T210, since both correlate negatively with BD (Li and Shao, 2006; Keller and Håkansson, 2010). However, vegetation has clearly influenced the BD on both transects since lateritic soils which lack any vegetative cover have been reported to have considerably higher bulk densities than that of our study site (Sherman et al., 1953). Plant roots, particularly those of woody species, help to maintain soil porosity and structure, thereby avoiding compaction as indicated by high BD (Li and Shao, 2006). The lower vegetation density on T140 would result in thinner, more compacted and eroded topsoils that are in closer proximity to, and therefore more greatly influenced by, the lateritic profile. The higher EC on T140 is also suggestive of greater erosion on this side of the hillslope since the pallid zone has an inherently high salt content, meaning that increased exposure of this horizon would elevate the salinity of surficial soils (Lewis, 1985; Brouwer and Fitzpatrick, 2002).

Although α-diversity did not vary significantly, it was consistently lowest in the upper sections which also had the highest EC values. The effects of salinity on bacterial α-diversity are still debated, with increased salinity causing reductions in species richness in some studies (Ibekwe et al., 2010; Xue et al., 2018), whilst having no significant effects in others (Gao et al., 2015). α-diversity has also been reported to be positively correlated with soil pH (Fierer and Jackson, 2006), which concurs with our results, as the upper sections had the lowest soil pH. β-diversity analysis indicated that, on both transects, the bacterial communities of the two most distant sections - the plateau and bottom - were more similar than those in-between. The bottom and plateau microbiomes exhibited positive associations with soil pH and sand content, while the upper sections correlated more with EC (on T140) and clay and MED content (T210). This concurs with Gleeson et al. (2016), who found that pH, EC, and MED were major driving factors, accounting for almost 60% of the variation in the bacterial community structure at a neighboring site within the AR-CZO. Our study therefore shows that soil heterogeneity (in terms of pH, EC, MED, and soil texture) relating to the different depths of the weathering profile is a major driver of compositional shifts in the soil microbial community. The significant PERMDISP result indicates that significant PERMANOVA results were also due to differences in the dispersion of bacterial communities between groups, in addition to compositional differences. However, the PERMANOVA results may also have been confounded by the variation in the number of samples per section which resulted from the failure of 5 DNA samples to pass the quality control filtering stages (Anderson and Walsh, 2013).

Relative to all other T140 sections, the T140 plateau was enriched in the phylum Gemmatimonadetes, in addition to several members of Proteobacteria and Actinobacteria. This may be related to this section having the highest water repellency (MED) on T140, since these phyla have been reported to be more abundant in water-limited environments (Acosta-Martinez et al., 2014; Curiel Yuste et al., 2014; Fernandes et al., 2018; Ren et al., 2018). For instance, Actinobacteria were enriched in the rhizospheres of drought-stressed rice (Santos-Medellín et al., 2017) and subterranean clover (Mickan et al., 2018), as well as water repellent soils of a Mediterranean semiarid forest (Lozano et al., 2014). This has been partially attributed to the wax-degrading ability of Actinobacteria (Roper, 2006), which corroborates our results since the dominant vegetation on T140 was *Eucalyptus*, the leaves of which contain epicuticular waxes (Wirthensohn and Sedgley, 1996; Doerr et al., 2000). This complements several other extreme environmental conditions that Actinobacteria species are known to tolerate, including high temperatures, salinity, acidity and alkalinity (Yandigeri et al., 2012; Mohammadipanah and Wink, 2016; Nguyen et al., 2018).

The prevalence of Actinobacteria in plateau samples may also be related to the weathering profile, since the iron oxide-rich duricrust layer was restricted to the plateau section of the hillslope. Actinobacteria are regarded as “subaerial settlers” that are well-adapted to hostile environments and dominate subaerial biofilms on exposed rock surfaces (Gorbushina, 2007). Indeed, Actinobacteria have been implicated in the bio-weathering of volcanic rocks in Iceland (Cockell et al., 2013) and granitic rocks in Egypt (Abdulla, 2009).

Several species enriched in the plateau and bottom sections are referred to as “rare” Actinobacteria as they produce bioactive compounds that have been used to develop novel antibiotics in the pharmaceutical industry (Lazzarini et al., 2001; Jose and Jebakumar, 2013; Azman et al., 2015). These include compounds with antibacterial, anticancer, nematocidal and antiviral properties (Tomita et al., 1993; Schupp et al., 1998; Ayar-Kayali and Tarhan, 2006; Igarashi et al., 2007; Ratnayake et al., 2007). The presence of these “rare” Actinobacteria was supported by our PICRUSt analysis, as the bottom sections of both transects had higher abundances of genes involved in the biosynthesis of penicillin, cephalosporin and ansamycins, which all have antibacterial and/or antitumor properties (Fleming, 1929; Neu, 1974; Rinehart and Shield, 1976; Basso et al., 2002). This site may, therefore, be suitable for future bioprospecting studies in search of novel actinobacterial taxa with valuable antimicrobial properties.

The T140 plateau was enriched in several families of Proteobacteria that contain atmospheric N-fixing species (Baldani et al., 2014; de Souza et al., 2014; Marín and Arahal, 2014). This was supported by the KEGG analysis which also indicated a greater abundance of N-fixing bacteria in the T140 plateau and bottom sections (Figure 8). N-fixing bacteria act as valuable biofertilizers that may be used either as supplements or alternatives to chemical fertilizers in sustainable agriculture (Ladha and Reddy, 1995; Monteiro et al., 2008; de Souza et al., 2015; Kumar et al., 2015). Therefore, if the soils in the paddock bordering our study site eventually inherit this abundance of N-fixers, it could benefit crop production. This increased abundance of N-fixers may result from the lower salinity of the plateau and bottom sections, corroborating reports that N-fixing bacteria abundance and activity is lower in saline soils (Sorokin et al., 2008; Thiem et al., 2018). It may also be related to the nutrient-poor status common to all surficial soils of aeolian origin, such as those covering the duricrust at the plateau which consist primarily of quartz sand and are therefore inherently nutrient deficient. *Beijerinckia* are well-adapted to lateritic soils, as they are able to grow in environments which lack calcium and have high levels of iron, which are common features of the upper layers of laterites (Becking, 1961a, b; Anand and Paine, 2002). Also, ferruginous gravels are known to have P-fixing attributes (Tiessen et al., 1991), which may mean that the plateau section has greater P content, thereby alleviating P-limitations on microbial N-fixation (Crews, 1993; Reed et al., 2007). The higher number of N-fixing bacteria detected in samples from the bottom section may be related to the potential influence of fertilizers applied to the paddock. However, chemical fertilizers have varying effects on N-cycling microbes, with inorganic N additions having no effect on *nifH* gene abundance in some studies (Berthrong et al., 2014; Sun et al., 2015), while others report a significant decrease in the number of *nifH* gene copies in bulk soil following inorganic (NH_4_^+^ and NO_3_^–^) N additions (Wang et al., 2017). A perhaps more likely driver for this is the significantly higher pH of the T140 plateau and bottom sections relative to the upper sections, as this would concur with Wang et al. (2017) who also reported a positive correlation between *nifH* gene copy number and soil pH.

Aspect appeared to influence the microbial communities of the upper sections only, with several taxa being significantly more abundant on T210 relative to T140 (Figure 7). We believe this was mainly caused by soil EC which was more than ten-fold higher in the upper sections of T140 than those of T210. EC is closely linked to several soil properties, notably salinity (Rhoades and Ingvalson, 1971), clay content (Sudduth et al., 2005), water content (Robinson et al., 2012), SOM (Martinez et al., 2009), and availability of total water-soluble ions, such as nitrate (Patriquin et al., 1993). Our finding that a member of the Acidobacteria (family *Solibacteraceae*) was significantly less abundant in the upper sections of T140 relative to those of T210 concurs with reports that Acidobacteria are negatively associated with soil EC (Wakelin et al., 2012). *Sphingomonas* abundance has also been shown to decline with increasing salinity, which complements our finding that *Sphingomonadaceae* were significantly less abundant in the upper sections of T140 (Yang et al., 2016). The increased abundance of *Actinomycetospora* on T210 upper sections may be driven by the higher water repellency on this transect, since species of this genus have previously been found in extremely arid environments, such as deserts (Sun et al., 2018). Certain members of *Sphingomonadaceae* also have associations with water repellent environments, as they can degrade polycyclic aromatic hydrocarbons which have hydrophobic properties (Leys et al., 2004). Microclimatic differences between aspects may also have directly or indirectly influenced microbial community composition (Timmusk et al., 2009; Wu et al., 2017), but at this relatively small spatial scale, we expect this to have a minor role in the differences observed in this study.

## Conclusion

Our study indicates the strong influence that weathering history, vegetation cover and their associated influences on soil physicochemical attributes can exert on the soil microbial community. The convergence of the microbial communities of opposite ends of both transects (i.e., the plateau and lower sections) appeared to be mainly driven by soil pH. This convergence suggests that the gradual erosion of this lateritic profile may eventually lead to an increased abundance of certain members of Proteobacteria and Actinobacteria as these were enriched in the plateau samples relative to the rest of the hillslope. Meanwhile, significant between-transect contrasts in bacterial community composition were limited to the upper sections. This appeared to be related to differences in SWR and EC, which we attribute to the greater exposure of the pallid zone in the T140 upper sections.

This enhanced understanding of the natural system could help to improve future management of agricultural systems in WA since they use soil with inherited physicochemical properties and bacterial communities. For instance, relative to the rest of T140, the plateau section was enriched in several families known to contain N-fixing bacteria. If the microbial communities of these laterite-derived agricultural soils continue to converge with those of the plateau over time via the weathering process, it could have important implications for agricultural management as it may reduce the need for inorganic N fertilizer applications.

## Data Availability

The datasets generated for this study can be found in Sequence Read Archive (NCBI), PRJNA494632.

## Author Contributions

ML visualized the concept for this study. MA, MF, AH, DH, EK, HL, M-AL, BM, VM, OM, FM, and FO’B carried out the fieldwork and analyzed the data. FM and FO’B processed the sequencing data. FM and FO’B interpreted the sequencing data. All authors contributed toward the writing of this manuscript with DG and ML helping in the overall structure.

## Conflict of Interest Statement

The authors declare that the research was conducted in the absence of any commercial or financial relationships that could be construed as a potential conflict of interest.

## Footnotes

1 https://www.ncbi.nlm.nih.gov/Traces/study/?acc=PRJNA494632

2 http://picrust.github.io/picrust/tutorials/genome_prediction.html

3 https://en.wikipedia.org/wiki/Corymbia_papuana
