# Supplementary material for: Soil Salinity and pH Drive Soil Bacterial Community Composition and Diversity Along a Lateritic Slope in the Avon River Critical Zone Observatory, Western Australia
Source: Front Microbiol. 2019 Jul 2;10:1486. doi: 10.3389/fmicb.2019.01486 (PMC6614384; doi:10.3389/fmicb.2019.01486)
Supplement: Supplementary file 1 [file Presentation_1.pptx]

## Slide 1
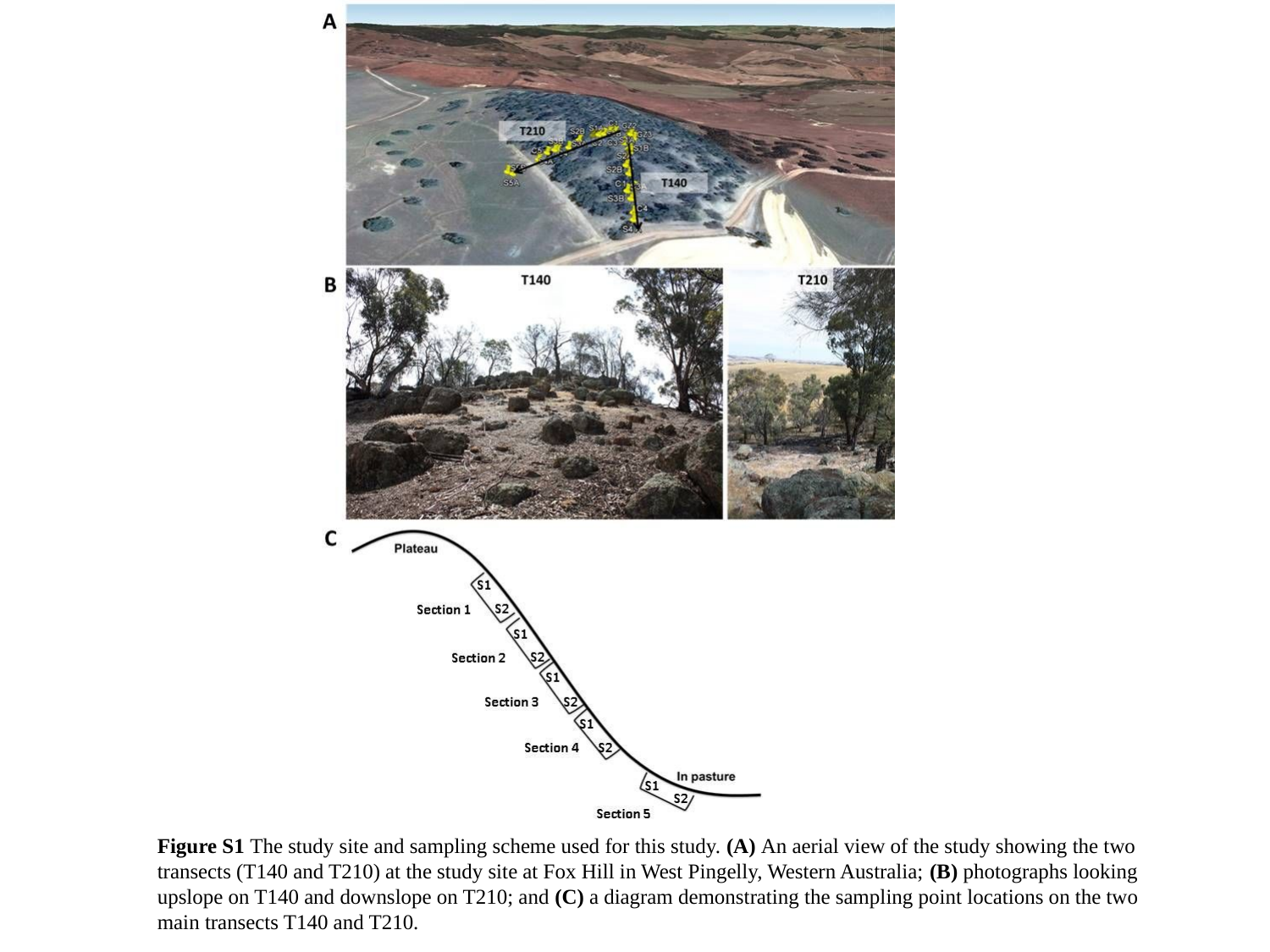

Figure S1 The study site and sampling scheme used for this study. (A) An aerial view of the study showing the two transects (T140 and T210) at the study site at Fox Hill in West Pingelly, Western Australia; (B) photographs looking upslope on T140 and downslope on T210; and (C) a diagram demonstrating the sampling point locations on the two main transects T140 and T210.

## Slide 2
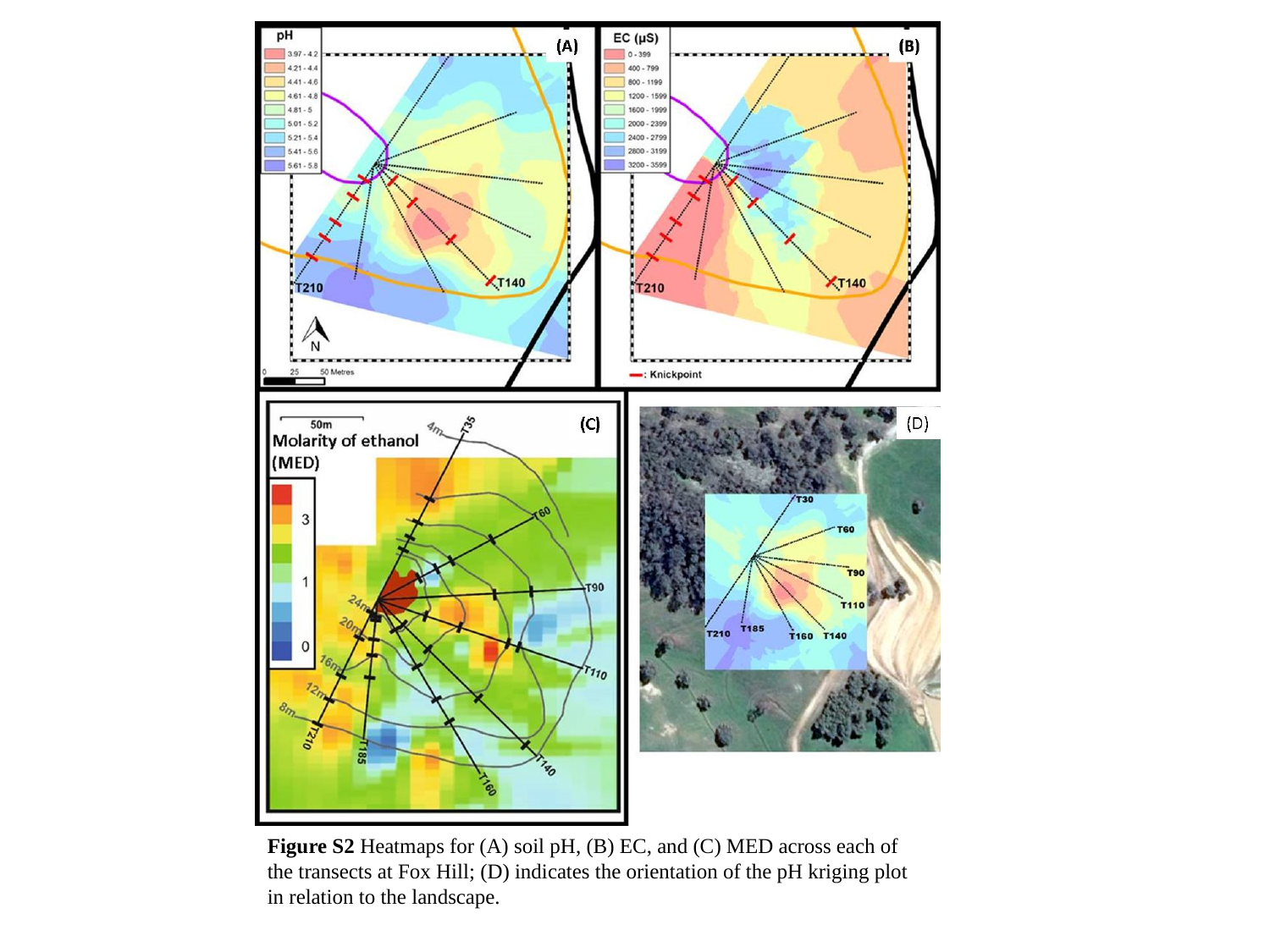

Figure S2 Heatmaps for (A) soil pH, (B) EC, and (C) MED across each of the transects at Fox Hill; (D) indicates the orientation of the pH kriging plot in relation to the landscape.

## Slide 3
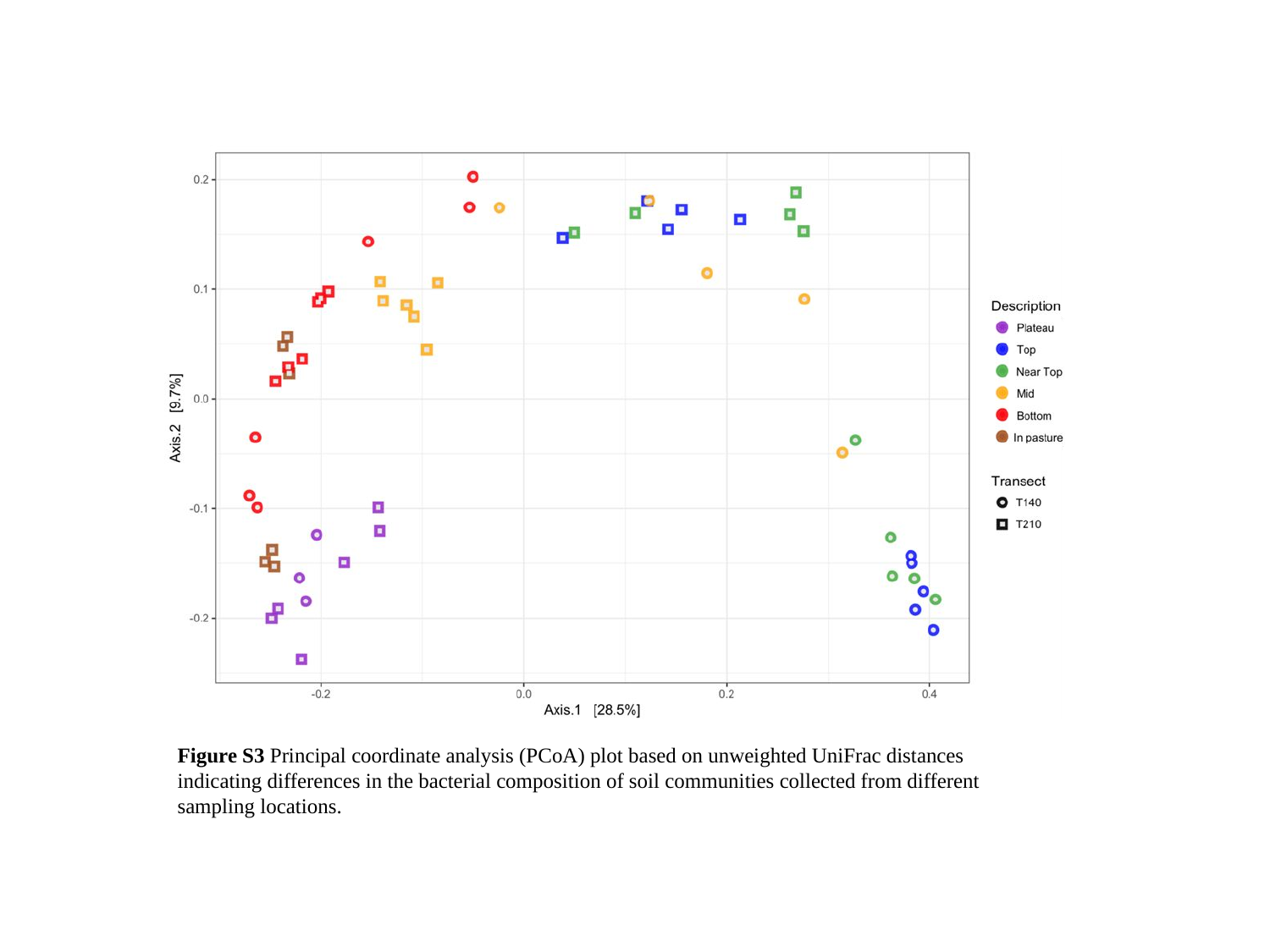

Figure S3 Principal coordinate analysis (PCoA) plot based on unweighted UniFrac distances indicating differences in the bacterial composition of soil communities collected from different sampling locations.

## Slide 4
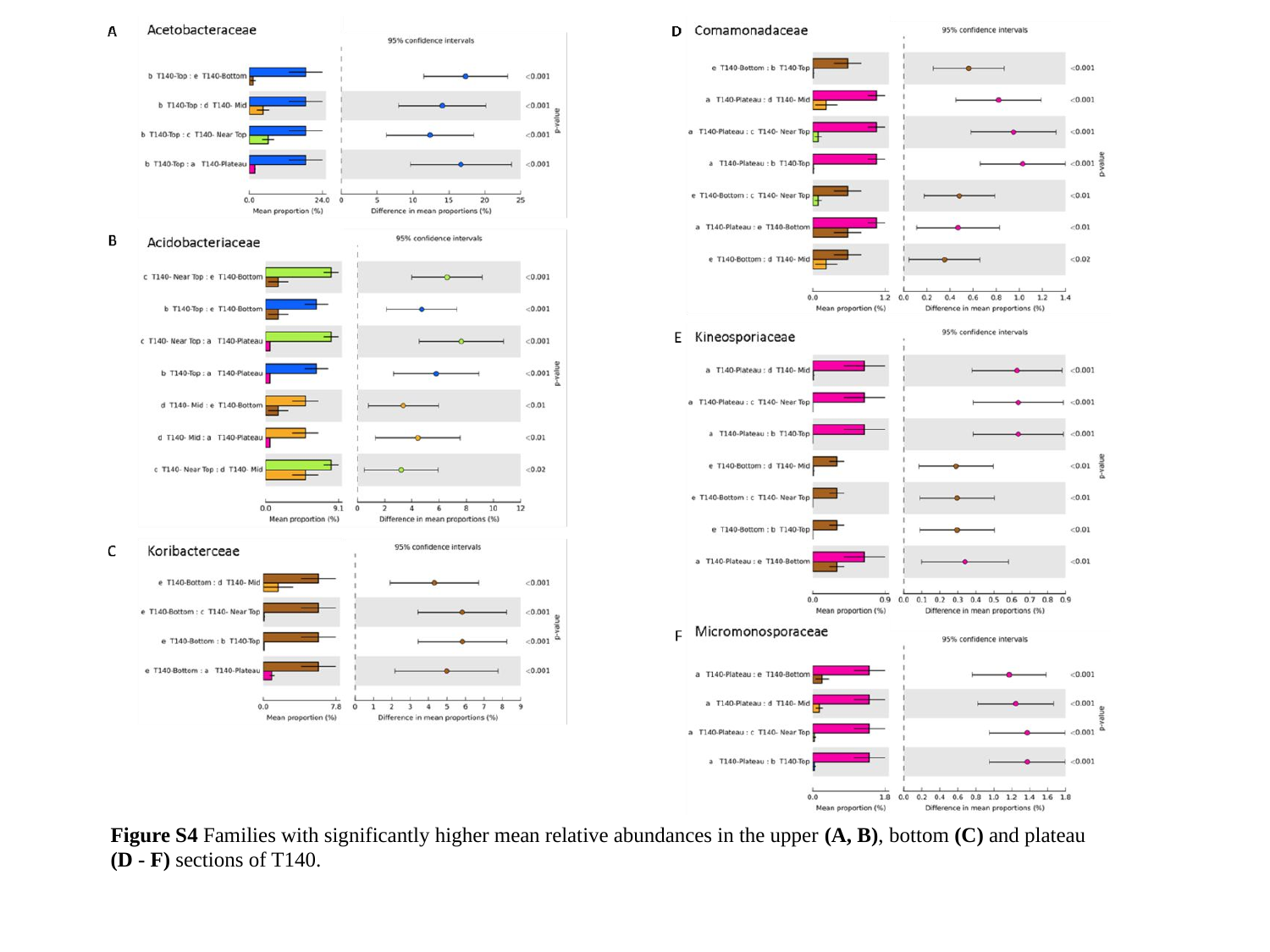

Figure S4 Families with significantly higher mean relative abundances in the upper (A, B), bottom (C) and plateau (D - F) sections of T140.

## Slide 5
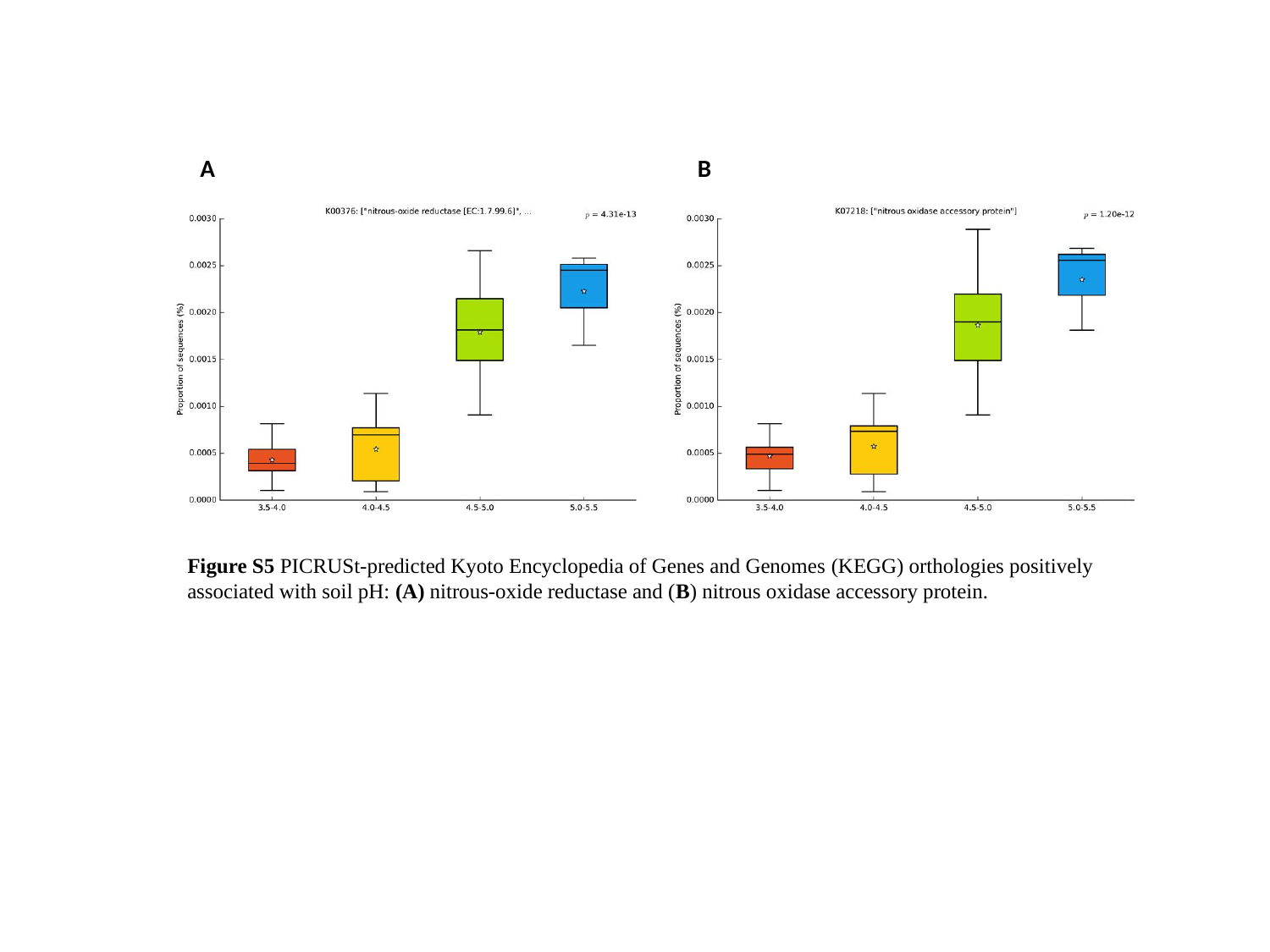

A
B
Figure S5 PICRUSt-predicted Kyoto Encyclopedia of Genes and Genomes (KEGG) orthologies positively associated with soil pH: (A) nitrous-oxide reductase and (B) nitrous oxidase accessory protein.
