# Supplementary material for: Soil Salinity and pH Drive Soil Bacterial Community Composition and Diversity Along a Lateritic Slope in the Avon River Critical Zone Observatory, Western Australia
Source: Front Microbiol. 2019 Jul 2;10:1486. doi: 10.3389/fmicb.2019.01486 (PMC6614384; doi:10.3389/fmicb.2019.01486)
Supplement: Supplementary file 2 [file Table_1.DOCX]

Table S1 Ranges used to create ranking groups of soil abiotic variables for multivariate analyses.

| **Measurement** | **Rank 1** | **Rank 2** | **Rank 3** | **Rank 4** | **Rank 5** | **Rank 6** | **Rank 7** | **Rank 8** |
| --- | --- | --- | --- | --- | --- | --- | --- | --- |
| pH | 3.5 - 3.39 | 4.0 - 4. 49 | 4.5 - 4.9 | 5.0 - 5.5 |  |  |  |  |
| MED (mol L^-1^) | 0-1 | 1-2 | 2-3 | 3-4 |  |  |  |  |
| EC (µS cm^-1^) | 0-50 | 50-100 | 100-150 | 150-200 | 200-250 | 350-400 | 800-1200 | > 1200 |
