# Supplementary material for: Soil Salinity and pH Drive Soil Bacterial Community Composition and Diversity Along a Lateritic Slope in the Avon River Critical Zone Observatory, Western Australia
Source: Front Microbiol. 2019 Jul 2;10:1486. doi: 10.3389/fmicb.2019.01486 (PMC6614384; doi:10.3389/fmicb.2019.01486)
Supplement: Supplementary file 3 [file Table_2.DOCX]

Table S2 Vegetation data for transects T140 and T210.

| **Plot ID** | **Description** | **Ground cover**  **(%)** | **Ground Cover:**  **Rank 1** | **Ground Cover:**  **Rank 2** |
| --- | --- | --- | --- | --- |
| GZ1 | T140-Plateau | 64.5 | 2 | 5 |
| T140S1A | T140-Top | 54.08 | 1 | 4 |
| T140S1B | T140-Top | 55.23 | 1 | 4 |
| T140S2A | T140-NearTop | 51.28 | 1 | 1 |
| T140S2B | T140-NearTop | 55.74 | 1 | 1 |
| T140S3A | T140-Mid | 47.94 | 1 | 1 |
| T140S3B | T140-Mid | 36.61 | 1 | 1 |
| T140S4A | T140-Bottom | 38.22 | 1 | 1 |
| T140S4B | T140-Bottom | 58.45 | 3 | 4 |
| GZ2,_T210P | T210-Plateau | 64.5 | 2 | 5 |
| T210S1A | T210-Top | 73.75 | 2 | 2 |
| T210S1B | T210-Top | 43.07 | 1 | 1 |
| T210S2A | T210-NearTop | 59.93 | 1 | 1 |
| T210S2B | T210-NearTop | 55.07 | 2 | 2 |
| T210S3A | T210-Mid | 46.48 | 2 | 4 |
| T210S3B | T210-Mid | 65.15 | 4 | 4 |
| T210S4A | T210-Bottom | 62.71 | 4 | 4 |
| T210S4B | T210-Bottom | 64.4 | 4 | 4 |

Ground Cover Species Type Key: 1- Leaf litter: *Eucalyptus salmonophloia* and *Eucalyptus papuana*; 2 - *Avena sativa* (spring oats); 3 - *Triticum* (wheat spp.); 4 - Native grass/forbs; 5 - Leaf litter: *Acacia* spp. Rank 1 represents the dominant vegetation type; and rank 2 the second-most dominant type.
