# Supplementary material for: Soil Salinity and pH Drive Soil Bacterial Community Composition and Diversity Along a Lateritic Slope in the Avon River Critical Zone Observatory, Western Australia
Source: Front Microbiol. 2019 Jul 2;10:1486. doi: 10.3389/fmicb.2019.01486 (PMC6614384; doi:10.3389/fmicb.2019.01486)
Supplement: Supplementary file 4 [file Table_3.DOCX]

Table S3 Alpha diversity metrics for samples grouped by sampling location (mean ± SD).

| **Sample Location** | **Observed species**  **(OTUs)** | **Chao1 index** | **Faith's phylogenetic**  **diversity** | **Shannon diversity**  **index** |
| --- | --- | --- | --- | --- |
| ***T140*** |  |  |  |  |
| Bottom | 753.93 ± 61.22 | 1289.09 ± 85.29 | 55.56 ± 4.21 | 8.74 ± 0.27 |
| Mid | 572.60 ± 127.80 | 975.45 ± 207.03 | 42.34 ± 8.38 | 7.87 ± 0.72 |
| Near Top | 456.92 ± 45.70 | 737.91 ± 92.49 | 34.00 ± 3.28 | 7.21 ± 0.32 |
| Top | 446.50 ± 42.10 | 675.73 ± 61.46 | 31.90 ± 3.03 | 7.48 ± 0.22 |
| Plateau | 749.80 ± 27.88 | 1257.72 ± 69.54 | 52.87 ± 2.97 | 8.83 ± 0.04 |
| ***T210*** |  |  |  |  |
| In Pasture | 746.97 ± 27.06 | 1292.86 ± 29.09 | 55.43 ± 0.94 | 8.64 ± 0.24 |
| Bottom | 697.47 ± 15.87 | 1173.70 ± 24.65 | 52.69 ± 1.03 | 8.54 ± 0.10 |
| Mid | 716.95 ± 27.19 | 1279.05 ± 76.42 | 53.96 ± 1.13 | 8.39 ± 0.11 |
| Near Top | 531.92 ± 79.13 | 901.84 ± 137.47 | 39.14 ± 6.32 | 7.60 ± 0.24 |
| Top | 578.40 ± 51.89 | 988.42 ± 122.36 | 42.47 ± 3.69 | 7.90 ± 0.24 |
| Plateau | 659.68 ± 60.52 | 1151.39 ± 136.94 | 47.47 ± 3.65 | 8.42 ± 0.28 |
