# Supplementary material for: Soil Salinity and pH Drive Soil Bacterial Community Composition and Diversity Along a Lateritic Slope in the Avon River Critical Zone Observatory, Western Australia
Source: Front Microbiol. 2019 Jul 2;10:1486. doi: 10.3389/fmicb.2019.01486 (PMC6614384; doi:10.3389/fmicb.2019.01486)
Supplement: Supplementary file 5 [file Table_4.DOCX]

Table S4 Pairwise PERMANOVA results comparing beta diversity of bacterial communities on the two principal transects grouped by sampling location using unweighted UniFrac distances. R^2^ values greater than 0.4 are highlighted in bold.

|  |  | **R^2^** | ***p-value* (FDR corrected)** |
| --- | --- | --- | --- |
| ***T140 comparisons*** | | | |
| **T140-Plateau** | **T140-Top** | **0.639** | 0.02 |
| **T140-Plateau** | **T140- Near Top** | **0.598** | 0.02 |
| **T140-Plateau** | **T140- Mid** | **0.424** | 0.024 |
| T140-Plateau | T140-Bottom | 0.250 | 0.02 |
| T140-Top | T140- Near Top | 0.285 | 0.013 |
| T140-Top | T140- Mid | 0.388 | 0.013 |
| **T140-Top** | **T140-Bottom** | **0.533** | 0.008 |
| T140- Near Top | T140- Mid | 0.306 | 0.013 |
| **T140- Near Top** | **T140-Bottom** | **0.495** | 0.01 |
| T140- Mid | T140-Bottom | 0.283 | 0.01 |
| ***T210 comparisons*** | | | |
| **T210-Plateau** | **T210-Top** | **0.429** | 0.008 |
| **T210-Plateau** | **T210-Near Top** | **0.422** | 0.018 |
| T210-Plateau | T210-Mid | 0.261 | 0.01 |
| T210-Plateau | T210-Bottom | 0.368 | 0.018 |
| T210-Plateau | T210-In pasture | 0.218 | 0.008 |
| T210-Top | T210-Near Top | 0.195 | 0.024 |
| T210-Top | T210-Mid | 0.313 | 0.018 |
| **T210-Top** | **T210-Bottom** | **0.466** | 0.008 |
| **T210-Top** | **T210-In pasture** | **0.419** | 0.01 |
| T210-Near Top | T210-Mid | 0.301 | 0.008 |
| **T210-Near Top** | **T210-Bottom** | **0.463** | 0.008 |
| **T210-Near Top** | **T210-In pasture** | **0.425** | 0.01 |
| T210-Mid | T210-Bottom | 0.308 | 0.008 |
| T210-Mid | T210-In pasture | 0.213 | 0.008 |
| T210-Bottom | T210-In pasture | 0.200 | 0.008 |
