# Supplementary material for: Soil Salinity and pH Drive Soil Bacterial Community Composition and Diversity Along a Lateritic Slope in the Avon River Critical Zone Observatory, Western Australia
Source: Front Microbiol. 2019 Jul 2;10:1486. doi: 10.3389/fmicb.2019.01486 (PMC6614384; doi:10.3389/fmicb.2019.01486)
Supplement: Supplementary file 6 [file Table_5.DOCX]

Table S5 Significant Tukey HSD results comparing group dispersion (PERMDISP) results.

| **Sample grouping** |  | ***P*-adjusted** |
| --- | --- | --- |
| **Sampling location (Transect & section)** | | |
| T140 Mid | T140 Plateau | 0.0359 |
| T140 Bottom | T140 Plateau | 0.0266 |
| T140 Mid | T140 Top | 0.0015 |
| T140 Bottom | T140 Top | 0.0008 |
| T210 Plateau | T140 Top | 0.0263 |
| T210 Near Top | T140 Top | 0.0183 |
| T210 Mid | T140 Top | 0.0448 |
| T140 Mid | T140 Near Top | 0.0261 |
| T140 Bottom | T140 Near Top | 0.0169 |
| T210 Bottom | T140 Bottom | 0.0334 |
| **pH rank** | | |
| 5.0-5.5 | 3.5-4.0 | 0.0010 |
| 5.0-5.5 | 4.0-4.5 | 0.0005 |
| 5.0-5.5 | 4.5-5.0 | 0.0007 |
| **EC rank** | | |
| 4 [150-200] | 1 [0-50] | 0.0006 |
| 4[150-200] | 3 [100-150] | 0.0014 |
| 6[300-400] | 4 [150-200] | 0.0034 |
| 7[800-1200] | 4 [150-200] | 0.0460 |
